# Role of intracellular water in the normal-to-cancer transition in human cells—insights from quasi-elastic neutron scattering

**DOI:** 10.1063/4.0000021

**Published:** 2020-09-08

**Authors:** M. P. M. Marques, A. L. M. Batista de Carvalho, A. P. Mamede, A. Dopplapudi, V. García Sakai, L. A. E. Batista de Carvalho

**Affiliations:** 1 Molecular Physical-Chemistry R&D Unit, Department of Chemistry, University of Coimbra, 3004-535 Coimbra, Portugal; 2 Department of Life Sciences, University of Coimbra, 3000-456 Coimbra, Portugal; 3 ISIS Facility, STFC Rutherford Appleton Laboratory, Chilton, Didcot OX11 0QX, United Kingdom

## Abstract

The transition from normal to malignant state in human cells is still a poorly understood process. Changes in the dynamical activity of intracellular water between healthy and cancerous human cells were probed as an innovative approach for unveiling particular features of malignancy and identifying specific reporters of cancer. Androgen-unresponsive prostate and triple-negative breast carcinomas were studied as well as osteosarcoma, using the technique of quasi-elastic neutron scattering. The cancerous cells showed a considerably higher plasticity relative to their healthy counterparts, this being more significant for the mammary adenocarcinoma. Also, the data evidence that the prostate cancer cells display the highest plasticity when compared to triple-negative mammary cancer and osteosarcoma, the latter being remarkably less flexible. Furthermore, the results suggest differences between the flexibility of different types of intracellular water molecules in normal and cancerous cells, as well as the number of molecules involved in the different modes of motion. The dynamics of hydration water molecules remain virtually unaffected when going from healthy to cancer cells, while cytoplasmic water (particularly the rotational motions) undergoes significant changes upon normal-to-cancer transition. The results obtained along this study can potentially help to understand the variations in cellular dynamics underlying carcinogenesis and tumor metastasis, with an emphasis on intracellular water.

## I. INTRODUCTION

Cancer is a worldwide health problem, being the second leading cause of death globally (9.6 million deaths in 2018) and expected to rise up to 22 million cases *per* year within the next two decades.^1^ An effective chemotherapy is therefore a pressing medical and social need, aimed at targeting neoplastic cells with minimal damage on healthy tissue. However, a successful development of novel anticancer agents relies on a thorough understanding of the carcinogenesis process, i.e., of the specific biochemical and biophysical mechanisms responsible for the normal-to-malignant transformation in cells that may enable both an early diagnosis and the development of selective and improved drugs. In particular, triple-negative breast adenocarcinoma is the most aggressive type of mammary cancer with a prevalence in younger women and with a very poor prognosis, for which little therapeutic progress has been achieved in the past decades.^2,3^ Prostate carcinoma is the most common cancer in men in western countries, the metastatic androgen-independent type being currently incurable.^4^ Osteosarcoma, in turn, is the most frequent bone malignancy in children and adolescents and the second most important cause of cancer-related deaths in this age group, with a limited prognosis regarding metastatic disease (survival < 20%).^5,6^

Tumor development results from uncontrollable cell growth due to cellular modifications, which are mainly triggered by DNA changes—a normal cell acquires new properties that enable it to proliferate independently, ultimately forming a tumor. This still largely unknown process is intimately related to the cell's biomechanical properties, which are dependent on the structural and dynamical behavior of the intracellular medium. Actually, intracellular water (the most abundant cell component) displays particular properties different from those of bulk water and is known to play a fundamental role in normal cell activity: through maintenance of the three-dimensional architecture and functional conformation of biopolymers (via their hydration layers) and by regulating vital biological processes (e.g., DNA transcription and translation for protein synthesis, energy generation, cellular signaling or neurotransmission).^7–9^ Since water provides the matrix in which all biochemical processes occur, its integrity (structural and dynamical) is fundamental for maintaining a healthy cellular state. Any alterations in its properties can be the driving force to disrupt homeostasis and initiate a series of events leading to cellular disfunction that can facilitate neoplastic growth. This role of water in the onset of disease is an innovative approach, which departs from the conventional “lock-and-key” interpretation of pathological states.^10^

Recently, attention has turned to the biophysics of the cancer state, shedding a new light on cancer beyond the recognized biochemical and genetic variations associated with malignancy, with a view to unravel the transition from healthy to cancer, as well as from localized tumors to metastatic states. Magnetic resonance imaging (MRI)^11,12^ and atomic force microscopy (AFM)^13–16^ studies evidenced significant differences in intracellular water between healthy and cancerous cells and revealed a strong correlation between malignancy and cellular plasticity, cancer cells being reported to display an enhanced deformability relative to healthy ones and invasive tumor cells being even “softer.” This increased flexibility associated with malignancy is supposed to allow neoplastic cells to grow uncontrollably (even in hostile microenvironments) and to contribute to their invasiveness and metastatic ability [epithelial-to-mesenchymal transition (EMT)].^17^ Unraveling cellular water behavior, at the molecular level, is therefore critical for determining how the biochemical and mechanical properties of cells are affected by the normal-to-cancer (NTC) transition, a dynamical non-equilibrium phenomenon that still remains poorly understood despite its undisputable impact on human health.

In the past decade, quasi-elastic neutron scattering (QENS) has been established as a technique of choice for the study of hydrogen-atom dynamics in biological systems, allowing to directly access different spatially resolved dynamic processes for key biological components—from fast localized modes to slower global motions—providing unique results not attainable by any other methods.^8,18–28^ Former studies by the authors have evidenced the feasibility of this technique to accurately probe both cytoplasmic and hydration water dynamics in cells and in hydrated biomolecules (e.g., DNA), particularly regarding the impact of anticancer drugs on the behavior of these types of interfacial water.^25–27,29^

Building on the success of these previous experiments, the present study applied QENS for probing the dynamical properties of water within neoplastic vs healthy human cells. This is a pioneer approach for unveiling particular features of malignantly transformed cells and attaining specific signatures of cancer, aiming at a better understanding of the normal-to-cancer transition at its earliest stage, which will hopefully allow a higher chemotherapeutic success. Furthermore, the knowledge thus gathered can help to elucidate the processes underlying cancer invasiveness and metastatic capacity, where other methods have failed. Since different tumors are known to display distinct chemical profiles (e.g., regarding protein or lipid composition and varying biomechanical features^30,31^), two distinct types of human cancer cells were probed—breast and prostate tissues—along with their non-tumorigenic (healthy) equivalents. The data were compared with results formerly obtained by the authors for osteosarcoma.^27^

## II. MATERIALS AND METHODS

The list of chemicals, as well as the complete experimental procedure for the preparation of the cell samples, is described in the supplementary material, together with details of the QENS data acquisition and analysis.

### A. Sample preparation

The following human cell lines were studied—cancer vs their non-tumorigenic counterparts (Table I): (i) triple-negative (metastatic) breast cancer (MDA-MB-231) and non-neoplastic mammary gland immortalized cells (MCF-12A)—hereafter named breast cancer and breast healthy, respectively; and (ii) androgen-independent prostate adenocarcinoma (PC-3) and normal prostate epithelium immortalized cells (PNT-2)—hereafter named prostate cancer and prostate healthy, respectively. The data were compared with the results formerly obtained by the authors for osteosarcoma (bone cancer).^27^

**TABLE I.** Designation used along the text for the human cell lines studied in the present work.

Cell line	Designation
MDA-MB-231	Breast cancer
MCF-12A	Breast healthy
PC-3	Prostate cancer
PNT-2	Prostate healthy
MG-63	Bone cancer (osteosarcoma)

The cells were grown on-site (at the Biology laboratory of the ISIS Pulsed Neutron and Muon Source of the Rutherford Appleton Laboratory), cultured as monolayers, at 37 °C in a humidified atmosphere of 5% CO_2_ (details in the supplementary material). Prior to the QENS measurements, the cell pellets were washed with deuterated phosphate buffered saline (PBS_deut_) and centrifuged in order to remove the extracellular water, following a previously optimized procedure (see the supplementary material).^25,27^ These experimental conditions ensure that, for all types of cells, the extracellular water was never more than 5% of the total water present, all the remaining being intracellular water (95%) and the interference from any extracellular water being negligible.

The values of the intracellular water mass-to-biomass ratio (weight of intracellular water vs weight of lyophilized cell pellet), expectedly different for each type of cell, were also determined: prostate healthy—19.5%; prostate cancer—24.2%; breast healthy—19.1%; breast cancer—20.2%; and osteosarcoma—18.6%. Nevertheless, these do not interfere with the interpretation of the QENS results, which is solely based on the dynamical behavior of intracellular water—the same for all cellular systems (95%).

### B. QENS

The QENS experiments were performed at the ISIS Pulsed Neutron and Muon Source of the Rutherford Appleton Laboratory,^32^ in the low-energy OSIRIS high-flux indirect-geometry time-of-flight spectrometer,^33,34^ which allows to probe water dynamics at picosecond timescales and on atomic lengths (details in the supplementary material).

All samples were analyzed at 310 K (to better represent physiological conditions). Data for the deuterated PBS buffer was also obtained, for comparison purposes. Elastic window scans were measured for all cell lines (in the temperature range of 10–310 K). A vanadium sample was also measured to define the instrument resolution and correct for detector efficiency.

Fitting of the QENS spectra was performed with the program DAVE [version 2.5, National Institute of Standards and Technology (NIST) Center for Neutron Research]^35^ (see the details in the supplementary material).

## III. RESULTS AND DISCUSSION

This study aimed to achieve detailed information on the ill-understood mechanisms underlying tumor initiation, progression, and metastasis. This type of cell transformation is associated with physiological, morphological, and molecular changes and is generally accepted that it is primarily induced by variations in DNA that trigger cells to proliferate uncontrollably. However, it is not clear how these changes are produced and how they accumulate in cells, particularly within the tumor microenvironment in the tissue matrix. Since the mechanical properties and water exchange kinetics of the intracellular milieu were found to be intimately associated with these processes,^11–16^ the dynamical profile of water in breast and prostate cancer cells was presently tackled by QENS and compared with their non-neoplastic (healthy) equivalents, as well as with bone cancer (osteosarcoma) cells.^27^

It should be emphasized that the different values of the intracellular water mass-to-biomass ratio determined for each type of cell under analysis do not interfere with the interpretation of the QENS results, since the dynamical profile of intracellular water is mainly determined by the water–water and water–biomass interactions and hence by the characteristics of the organelles/membranes/biomolecules present, but not by the quantitative relationship water mass-to-biomass. Actually, although this ratio may be distinct for the distinct cells under study, the water's molar fraction is so much larger relative to all other cellular components that those differences are not expected to interfere with the QENS results. Additionally, the contributions from water within the cell (cytoplasmic or hydration water) were clearly differentiated upon a careful analysis of the experimental data currently obtained.

**FIG. 1.**
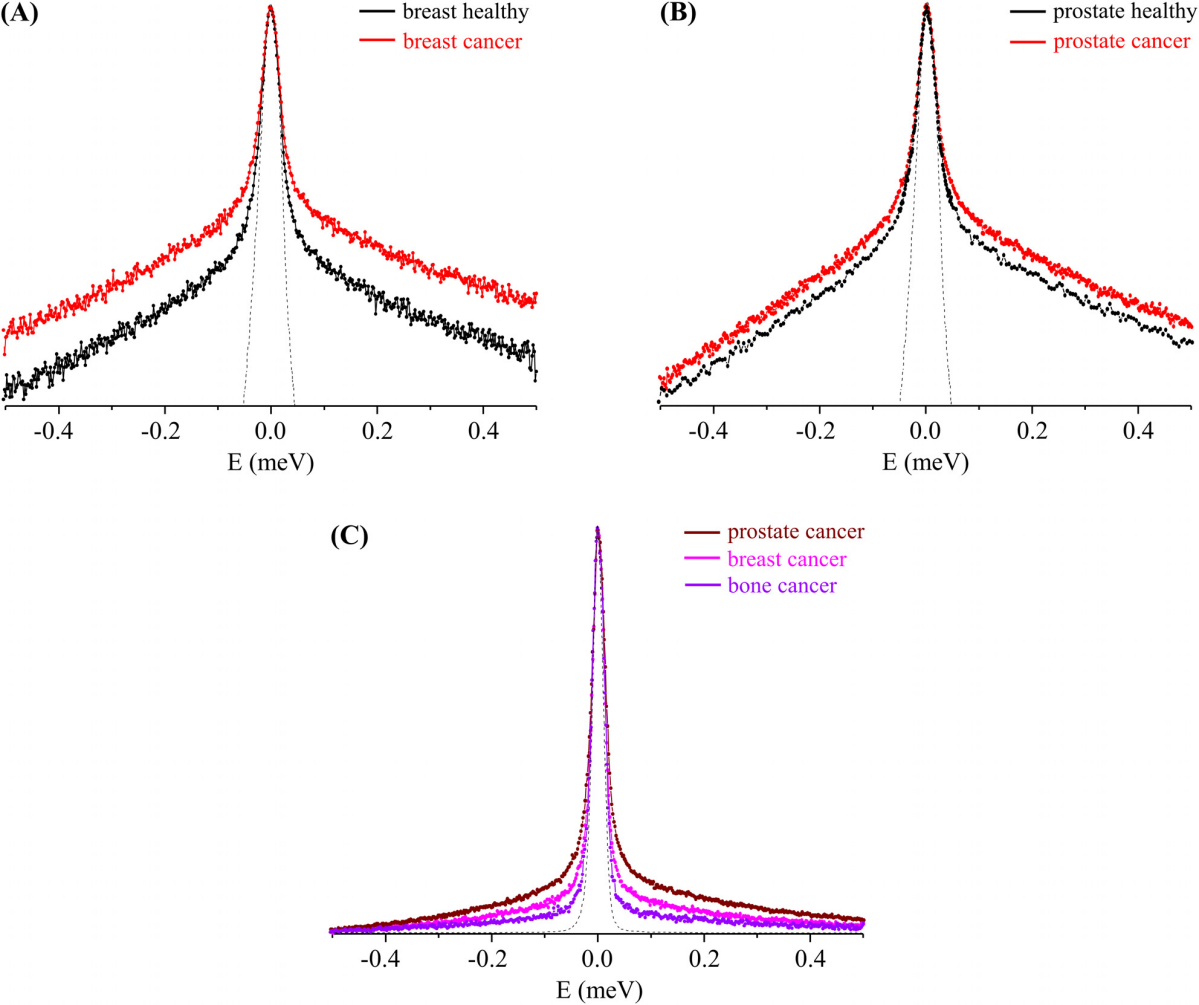
QENS profiles (310 K, at Q = 1.684 Å^−1^) for cancer and healthy human cells: (a) breast cancer and breast healthy, (b) prostate cancer and prostate healthy, and (c) all cancer cells studied—breast, prostate, and bone (the spectra were normalized to the maximum peak intensity. The dashed line represents the instrument resolution, as measured by a standard vanadium sample. The QENS profiles (a) and (b) are represented in the *yy*´ logarithmic scale).

The QENS profiles obtained for these types of tumor and non-tumor cellular models clearly evidenced a higher flexibility of the former relative to the healthy ones, this difference being much more significant for the breast cells as compared to that for the prostate ones [Fig. 1(a) vs Fig. 1(b)].

Furthermore, for the healthy cells, the dynamical behavior varied according to the nature of the tissue where the cancer originates—either breast and prostate cancers or bone cancer (previously studied^27^)—the latter having been shown to display a remarkably lower overall flexibility followed by metastatic breast and prostate cancers [Fig. 1(c)]. This is probably related to the different tumor histology—cancers from the epithelial tissue (e.g., breast and prostate), as compared to sarcomas, from the connective tissue (e.g., bone cancer)—and is in line with the biochemical differences already observed by the authors for these systems [using Raman, Fourier transform infrared (FTIR), and nuclear magnetic resonance (NMR) spectroscopies].^27,36–39^

**FIG. 2.**
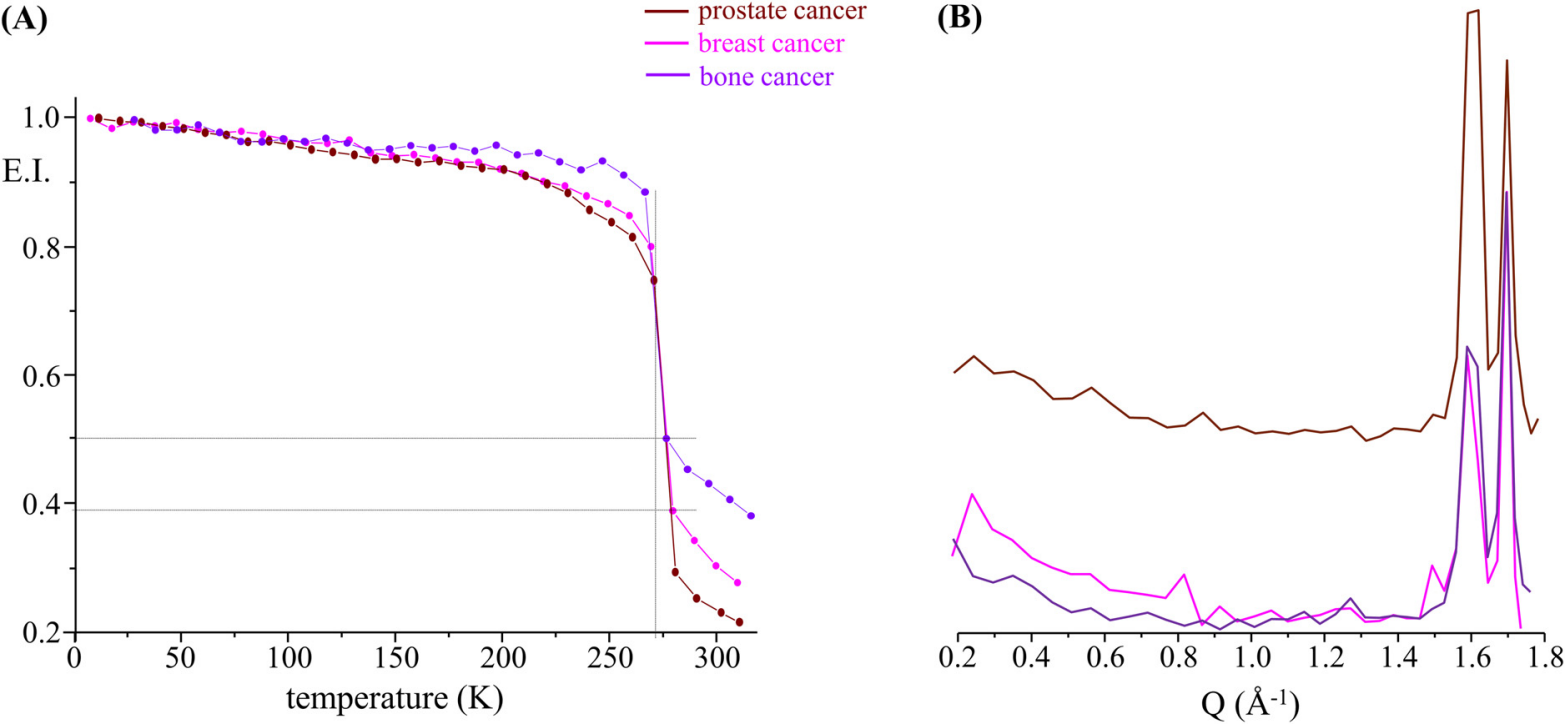
Elastic scan plots as a function of T (a) and of Q (at 270 K) (b), for all cancer cells studied—breast, prostate, and bone cancers [in (a), the plots represent the elastic intensity integrated over the OSIRIS instrumental resolution, normalized to the elastic intensity obtained at the lowest temperature (10 K)].

A common measurement in the QENS technique involves monitoring the elastic intensity as a function of temperature over a broad range of temperatures (elastic fixed window scan, EFWS), here from 10 to 310 K. This type of measurement gives an indication of the overall cellular microscopic dynamics, allowing us to locate dynamical transitions and highlighting structural rearrangements within the system, both of which may help to better differentiate non-malignant from cancer cells, as well as different types of carcinomas. Elastic fixed window scans for the cancerous and healthy cell samples presently studied revealed some noteworthy signatures (Fig. S1, supplementary material). At very low temperatures, the measured intensity is predominantly elastic, as the system is mostly immobile on the timescale of the spectrometer; as the temperature is raised, in turn, atoms can vibrate and start moving until eventually a dynamical transition takes place at *ca.* 270 K, reflecting the melting of ice within the intracellular milieu. The viscous nature of the samples (cell pellets, without extracellular water) justifies the quasi-horizontal decline in elastic intensity from base temperature up to this transition point. A certain degree of hysteresis was observed for all samples, *ca.* 270 vs 245 K for heating and cooling, respectively (data not shown), expected for melting and crystallization. In addition, a comparison between the samples shows slight differences between the elastic intensity prior to ice melting, suggestive of differences in the frozen structures. Qualitatively, bone cancer appears to form a much more rigid frozen structure than the other two cancerous cell lines (breast and prostate). This is shown in Fig. 2(a), which depicts the elastic intensity plotted as a function of T for all tumor lines. To get a better insight into the structural configuration of the systems, the elastic intensity can also be plotted as a function of Q (at 270 K in the frozen state) [Fig. 2(b)]. It should be noted that this type of measurement does not provide a very detailed structural picture, but rather a coarse qualitative information. The results shown in Fig. 2(b) evidence clear differences in the Q-dependence of the elastic intensity. Most significant are the visible variations in the intensity of the Bragg peaks observed at *ca.* 1.6 and 1.7 Å^−1^, corresponding to distances of *ca.* 3.9 Å and 3.7 Å, respectively, which are a signature of hexagonal ice and thus suggest structural differences in the crystal network. Similar observations were made between healthy and malignant cells, as well as among the distinct types of cancer currently investigated (Fig. S2, supplementary material), which is further evidence of the biochemical, morphological, and biophysical dissimilarities between them. In the light of the present results, the prostate cancer cells show the most significant variations when compared to osteosarcoma and triple-negative mammary carcinoma.

**FIG. 3.**
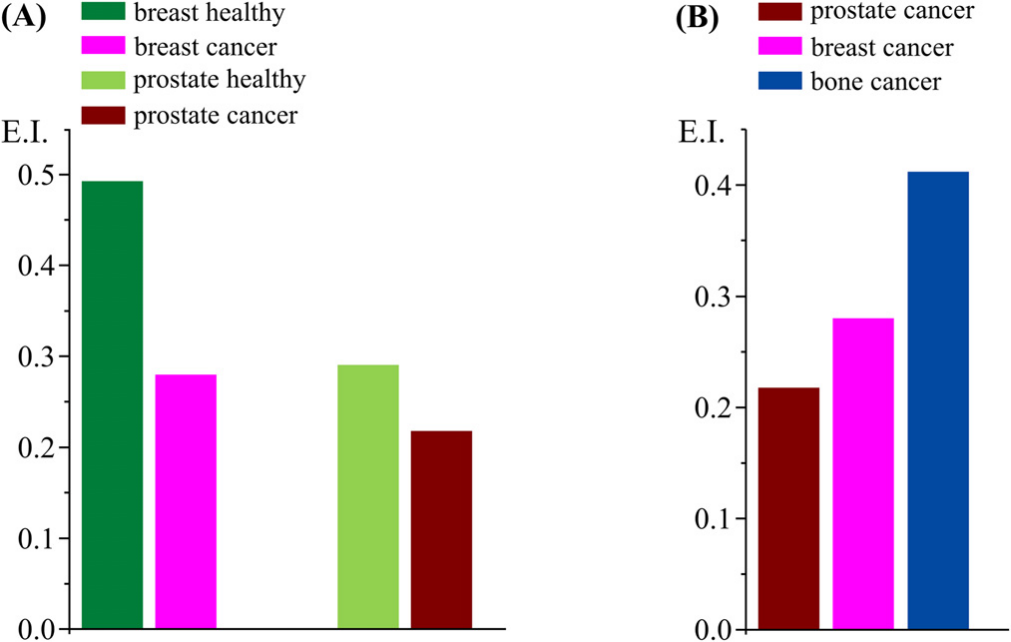
Plots of elastic intensity at 310 K, for cancer and healthy human cells: (a) breast healthy vs breast cancer, and prostate healthy vs prostate cancer; (b) all cancer cells studied—breast, prostate, and bone.

The third and final signature is the elastic intensity at the physiological temperature of 310 K. A marked difference between cancer and non-cancer cells (both breast and prostate) is observed, suggesting that the malignant systems contain more mobile species (in the OSIRIS timescale) than the healthy cells. In addition, when comparing all tested malignant cells [Figs. 2(a) and 3], prostate carcinoma shows the highest plasticity compared to breast and bone cancers, in that order [Fig. 3(b)], corroborating the respective QENS profiles [Fig. 1(c)]: while prostate carcinoma showed the softest character, osteosarcoma was the most rigid cellular matrix currently analyzed.

Significant chemical changes, leading to an enhanced cell proliferation and motility, have been related to cancer development, malignancy degree, and invasiveness—namely, the fatty acid profile (e.g., increased unsaturation degree) in some invasive tumor cells such as the triple-negative breast cancer currently investigated.^31,40^ These noticeable biochemical differences may underlie distinct cellular dynamical profiles and an altered expression of enzymes (e.g., tyrosine kinases and fatty acid synthase), adhesion molecules, and structural proteins (e.g., from the cytoskeleton, membrane-bound, and collagen).^30,41^ Furthermore, the greater plasticity of neoplastic vs healthy cells is proposed by some authors to be associated with their aggressive potential—invasiveness and metastatic ability^12,31,42^—which is considerably higher for prostate cancer and metastatic breast cancer (lacking the estrogen, progesterone, and human epidermal growth factor receptors), as compared to the poorly metastatic bone cancer (osteosarcoma). The results presently obtained are in accordance with this scenario, the prostate carcinoma cells displaying the highest mobility followed by breast cancer, while osteosarcoma cells were shown to be the least flexible system [Figs. 1(c) and 3(b)]. Moreover, an increased motility of the cellular matrix was observed upon normal-to-malignant transformation, as clearly reflected in Figs. 1(a), 1(b), and 3(a).

Discrimination of the distinct dynamical processes taking place within heterogeneous cellular matrices relies on an accurate fitting of the experimental results. This was accomplished using one δ-function (elastic component) convoluted with the line shape of the instrument and Lorentzian functions to represent the quasi-elastic contributions, according to the model previously optimized by the authors for breast carcinoma cells^25^ and already applied successfully to represent microscopic diffusion processes in osteosarcoma cells^27^ and other biological systems (such as living planarians^43^). Actually, as previously found,^25,27^ these are too complex systems to be accurately reproduced with only two Lorentzian functions (Γ_global_ and Γ_local_): apart from Γ_local_ that characterizes the fast localized dynamics of the biomolecules (e.g., DNA, proteins, and lipids, which cannot be discriminated) and the rotation of cytosolic water, two other Lorentzians are required to represent the intracellular water molecules, which have distinct dynamical regimes depending on their location—either in the cytoplasm (with a higher mobility, Γ_global/cyt_) or in the constrained hydration layers around biomolecules (Γ_global/hyd_). Accordingly, the following dynamical components were considered: (i) very slow motions from the biomass (slower than the longest observable time defined by the spectrometer resolution)—largest organelles and cytoskeleton and global motions of the macromolecules, represented by a Delta function; hence, this elastic line encompasses the cellular constituents with a very slow dynamics, but not water; (ii) slow diffusion of the intracellular water molecules (Q-dependent reorientations mediated by hydrogen bonds), both for the cytoplasmic and hydration water—defined by two Lorentzian functions for each of the two types of intracellular water (Γ_global_); (iii) internal localized motions (Q-independent), comprising conformational rearrangements from the biomolecules and lipid motions such as the lateral diffusion of phospholipids (at 310 K) and the movement of cholesterol within the membranes—ascribed to a broader Lorentzian (Γ_local_) (Fig. S3, supplementary material).

**FIG. 4.**
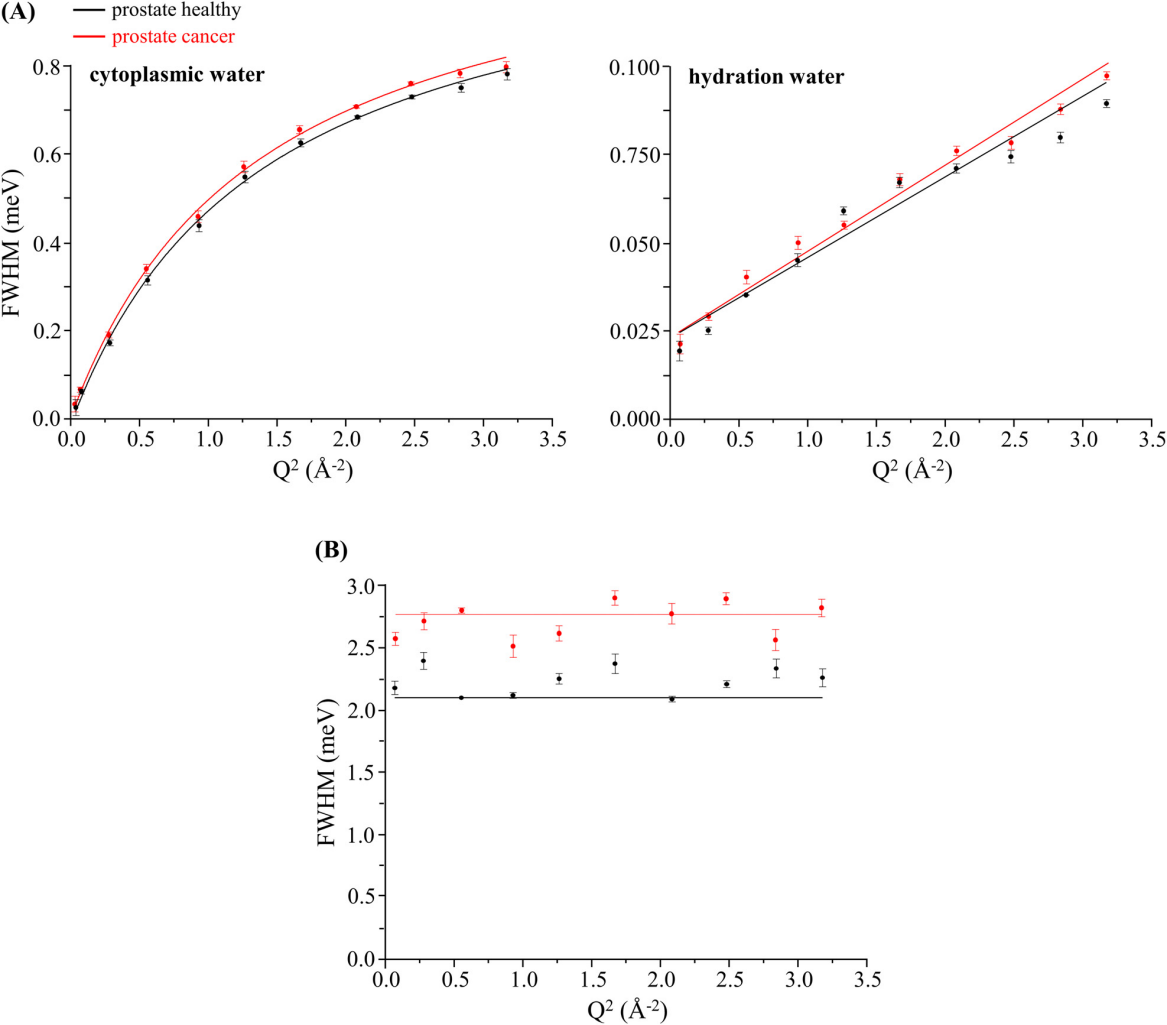
Variation of the full widths at half-maximum (FWHM) with Q^2^ for human prostate cancer and prostate healthy cells (at 310 K): (a) Lorentzian functions representing the translational motions of intracellular water—cytoplasmic medium and hydration layers (Γ_global_); and (b) Lorentzian function representing the internal localized motions within the cell (Γ_local_).

Despite the obvious difficulty of specifically assigning all the dynamical components in such a heterogeneous and complex system as a human cell, a feasible representation was achieved, allowing to obtain diffusion coefficients (D_T_) and residence times (τ_T_). Figure 4 depicts the Q-dependence of the full width at half-maximum (FWHM = Γ) of the Lorentzian functions representing the cellular dynamical constituents, for the prostate cells presently studied (cancer and healthy): the cytoplasmic and hydration water dynamics displaying a Q-dependent behavior and the faster localized motions exhibiting a Q-independent profile. As expected, cytoplasmic water was found to be more flexible than the highly organized hydration layers, its dynamics being well represented by a non-diffusive jump reorientation model^44–46^ [Γ_global_ asymptotically increasing to a plateau; Eq. (5) of the supplementary material]. Regarding hydration water, a restricted Fickian diffusion was evidenced [Fig. 4(a)].

The diffusion coefficients and relaxation times obtained for the systems under study revealed distinguishing features between non-tumor and tumor cells, quantitatively reflecting their distinct dynamical behavior (in the picosecond timeframe; Table II). It was confirmed that the flexibility of the cytomatrix is enhanced in malignant cells as compared to that in healthy ones—D_T_ increases by *ca.* 5% and 11%, respectively, in prostate and breast cancers, while the corresponding τ_T_ values decrease by *ca.* 2.3% and 0.9%. A similar trend was evidenced for the hydration layers surrounding the cellular macromolecules. Regarding the correlation times for the faster localized motions of the cellular constituents (represented by Γ_local_), the residence times showed a decrease upon malignant transition also evidencing a faster dynamics, which is particularly noteworthy for prostate carcinoma—*ca.* 33% as compared to 5% for breast cancer. This enhanced mobility in malignant cells can be justified by the highest plasticity of their intracellular medium, which may ease the localized motions of the macromolecular solutes in the crowded cytoplasmic environment in which they move.

**TABLE II.** Translational diffusion coefficients (D_T_) and relaxation times (τ_T_, τ_L_) of intracellular water (at 310 K) for cancer and non-cancer human cells: breast cancer vs breast healthy; prostate cancer vs prostate healthy; bone cancer [slow global translational (Γ_global/cyt_ and Γ_global/hyd_) and fast localized (Γ_local_) dynamical processes].

Cell line	Γ_global/cyt_		Γ_global/hyd_		Γ_local_
	D_T_ (×10^−5^ cm^2^ s^−1^)	τ_T_ (ps)	D_T_ (×10^−5^ cm^2^ s^−1^)	τ_T_ (ps)	τ_L_ (ps)
Breast cancer	1.126 ± 0.048 ^a^1.040 ± 0.050	0.597 ± 0.018	^b^0.169 ± 0.004	7.021 ± 0.222	0.561 ± 0.001
Breast healthy	1.014 ± 0.060	0.602 ± 0.026	^b^0.151 ± 0.019	7.353 ± 0.435	0.589 ± 0.006
Prostate cancer	1.308 ± 0.040	0.559 ± 0.011	^b^0.210 ± 0.016	6.475 ± 0.215	0.362 ± 0.0352
Prostate healthy	1.245 ± 0.043	0.572 ± 0.013	^b^0.179 ± 0.003	6.654 ± 0.154	0.480 ± 0.003
Bone cancer	0.908 ± 0.061	0.629 ± 0.038	^b^0.127 ± 0.016	7.699 ± 0.496	0.449 ± 0.015

^a^ At 298 K, from Ref. 25.

^b^ Fickian behavior (Γ = 2DQ^2^).

**FIG. 5.**
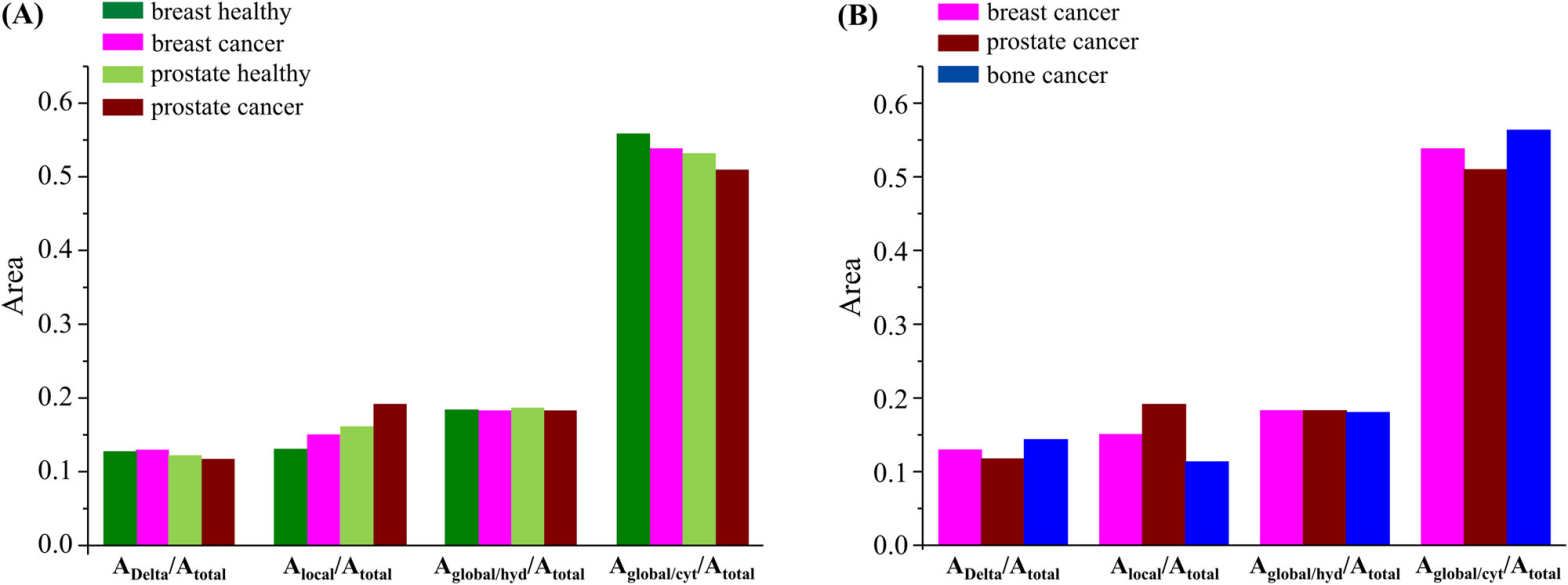
Area ratios for the different functions that characterize the dynamical behavior of intracellular water (at 310 K) in the human cells under study, for Q = 1.124 Å^−1^—Lorentzian functions representing the localized rotations (Γ_local_) and translational motions of cytoplasmic and hydration intracellular water (Γ_global/cyt_ and Γ_global/hyd_): (a) breast healthy vs breast cancer, and prostate healthy vs prostate cancer; (b) all cancer cells studied—breast, prostate, and bone (the data are plotted at a representative intermediate Q-value; however, the trend is similar across the bank).

Figure 5 represents the amplitudes of the different components used to characterize the dynamical behavior of intracellular water—one Delta (very slow motions that may be considered as immobile components in the OSIRIS timeframe) and three Lorentzians (Γ_global/cyt_ and Γ_global/hyd_ for the translations and Γ_local_ for the faster rotations). These plots can give an insight into the relation between immobile vs mobile species in the healthy and cancerous cellular systems and into the effects of malignancy on the various dynamical motions taking place. The contribution of the Delta function is rather similar for all four samples, evidencing that the biomolecular composition and dynamical behavior are similar between cells and between healthy and cancerous systems. In terms of the dynamical processes, the mobility differences between malignant and non-malignant samples (both for prostate and breast) are visible in the contributions of the local dynamics and of the global/cytoplasmic water, thus revealing a significant contribution from the localized rotational processes and the translations within the intracellular milieu. Since the different types of cancer cells under study are known to have different chemical profiles, namely, regarding protein or lipid composition (e.g., for triple-negative breast cancer, the cellular membrane is recognized to be highly flexible due to an increased unsaturation of its phospholipids), it is not surprising that the localized dynamical components (which encompass motions from proteins, RNA, DNA, and lipids) play such an important role in discriminating tumor from non-tumor cells. Interestingly, the extent of localized rotational motions is always higher in the cancerous cells (which may be related to their higher plasticity), but the contribution of water molecules diffusing in the cytoplasm is smaller. In contrast, the translational motions of hydration water do not appear to change much between non-tumor and tumor cells, which seems to reveal that the hydration layers around biomolecules are kept mostly unchanged. When comparing the three types of cancer cells currently probed, the relationships displayed in Fig. 5(b) clearly show the higher input from the local dynamical components (Γ_local_) for discriminating between these malignant cellular systems, as well as the contribution from the translational motions of cytoplasmic water (Γ_global/cyt_), whereas the dynamics within the biomolecules' hydration layers are virtually unchanged. Moreover, the immobile-to-mobile ratio is larger for bone cancer, in agreement with the corresponding QENS profiles.^27^ These conclusions can be quantified through the ratios obtained for the distinct dynamical components within the cellular matrices, namely: A_Delta_/A_(Γglobal+Γlocal)_ (at Q = 1.124 Å^−1^)—0.132 (prostate cancer) vs 0.138 (prostate healthy), and 0.132 (prostate cancer) vs 0.148 (breast cancer) vs 0.167 (bone cancer); A_Delta_/A_(Γglobal)_ (at Q = 1.124 Å^−1^)—0.169 (prostate cancer) vs 0.170 (prostate healthy), 0.169 (prostate cancer) vs 0.179 (breast cancer) vs 0.193 (bone cancer). In addition, the A_local_/A_total_ relationships [Fig. 5(a)] follow the values of the intracellular water mass-to-biomass ratios currently determined for the different cells under study—water-to-biomass increasing for the malignant vs the corresponding healthy cells.

The data presently obtained may be compared with former measurements performed by the authors on the impact of anticancer drugs on intracellular water (in the same timeframe): while drug-exposure induced a decreased flexibility of the cytoplasmic medium along with a disruption of the biomolecules´ hydration layers,^25,26,29^ normal-to-cancer transition was found to be accompanied by an increasing plasticity of the cytomatrix. These combined results corroborate the key role of intracellular water dynamics in the cell proliferation and differentiation processes: (i) regarding the drugs´ mode of action, the perturbation of water's dynamical profile mediates cytotoxicity and leads to cell growth-inhibition/death; and (ii) concerning normal-to-malignant transformation, water dynamics may be closely associated with the onset of unchecked cell growth (carcinogenesis) and invasiveness.

## IV. CONCLUSIONS

The current study aimed at shedding new light into the poorly known process of normal-to-cancer (NTC) transformation. It went a step further regarding previous experiments by the authors: (i) through vibrational microspectroscopy (FTIR and Raman), providing data on metabolic variations among distinct types of cancer cells and changes between drug-treated and untreated malignant cells,^27,36,37^ as well as through NMR metabolomics^38,39^ and (ii) by QENS, evidencing differences in the dynamical profile of drug-free and drug-exposed human malignant cells.^25,26,29^ The cellular biomechanical properties were tackled with an emphasis on the impact of intracellular water dynamics in oncogenic transformation and tumor aggressiveness, constituting further evidence of the recognized dissimilarities between healthy and cancer cells as regards the morphological, biochemical, and functional properties. The dynamical profiles obtained for cancer vs non-malignant cells showed a clear discrimination between them, tumor cells displaying a significantly higher plasticity relative to the non-cancer ones, an enhanced mobility of intracellular water thus being a potential hallmark of malignancy. Furthermore, the tumor histological nature was found to be associated with a different cellular dynamics—namely, prostate, breast, or bone cancers, the latter having revealed a remarkably lower flexibility, while the former showing the highest plasticity. Interestingly enough, the water molecules within the biomolecules´ hydration layers seem to remain unaffected by either healthy or malignant cells, or by cancer type, cytoplasmic water and mostly the rotational motions of water evidencing the most noticeable variations from non-malignant to cancer systems. Elucidation of this effect requires further studies, which are foreseen for different types of human cells (cancerous and non-cancerous).

In conclusion, apart from the known biochemical/metabolic differences between cancer and healthy human cells, the present results evidenced unequivocal biomechanical changes, namely, in the respective intracellular water dynamics, which can be regarded as a specific reporter of the cellular state. In addition, they allowed to differentiate the several dynamical components within these systems and to characterize them according to the nature of the tissue and the type of cancer. Despite this study containing a very limited number of samples, this physico-chemical description of malignancy is expected to allow a better understanding of the origins of cancer and of the processes underlying its progression, leading to more effective diagnosis and to the development of improved therapeutic strategies.

## SUPPLEMENTARY MATERIAL

See the supplementary material for the list of chemicals, the complete experimental procedure for the preparation of the cell samples, and details of the QENS data acquisition and analysis.
The supplementary material also contains Figs. S1, S2, and S3: Fig. S1—elastic scan plots (10–310 K) as a function of T, for cancer and healthy human cells: (a) breast cancer vs breast healthy and (b) prostate cancer vs prostate healthy [the plots represent the elastic intensity integrated over the OSIRIS instrumental resolution, normalized to the elastic intensity obtained at the lowest temperature (10 K)]. Figure S2—Temperature variation of the mean square displacements (10–310 K), for cancer and healthy human cells: (a) breast cancer vs breast healthy cells, (b) prostate cancer vs prostate healthy cells, and (c) prostate cancer vs breast cancer cells [the plots represent the elastic intensity integrated over the OSIRIS instrumental resolution, normalized to the elastic intensity obtained at the lowest temperature (10 K)]. Elastic scan plots as a function of Q: (d) breast cancer vs breast healthy cells, at 270 K and (e) prostate cancer vs prostate healthy cells, at 270 and 280 K. Figure S3—QENS spectra (310 K) for human prostate cancer and prostate healthy cells, fitted using three Lorentzians and one Delta functions, at some typical Q values.

## AUTHORS' CONTRIBUTIONS

M.P.M.M.—conceptualization, experimental measurements, data analysis, and manuscript writing; A.L.M.B.d.C. and A.P.M.—sample preparation, experimental measurements, and formatting of final manuscript; A.D.—assistance in sample preparation (cell culture, at ISIS Facility labs); V.G.S.—experimental measurements, data analysis, and manuscript writing; and L.A.E.B.d.C.—experimental measurements. All authors have read and agreed to the published version of the manuscript.

## Data Availability

The data that support the findings of this study are available from the corresponding author upon reasonable request.
